# *AC010883.*5 promotes cell proliferation, invasion, migration, and epithelial-to-mesenchymal transition in cervical cancer by modulating the MAPK signaling pathway

**DOI:** 10.1186/s12885-023-10825-2

**Published:** 2023-04-21

**Authors:** Qiyu Gan, Xia Huang, Wenrong Zhao, Hui Liu, Yan Xu, Xiaohua Zhang, Jingxin Cheng, Rui Chen

**Affiliations:** 1 Department of Gynecology and Obstetrics, School of Medicine, Shanghai East Hospital, Tongji University, Shanghai, 200120 China; 2 Department of Gynecology and Obstetrics, Shanghai East Hospital Ji’an Hospital, 80 Ji’an South Road, Ji’an City, 343000, Jiangxi, China; 3 Department of Pathology, School of Medicine, Shanghai East Hospital, Tongji University, Shanghai, 200120 China; 4 Department of Gynecology, United Family Hospital, Shanghai, China

**Keywords:** *AC010883.5*, Cervical cancer, MEK1/2, ERK1/2, MAPK signaling pathway

## Abstract

Homo sapiens chromosome 2 clone RP11-339H12 (*AC010883.5*) is a dysregulated long non-coding RNA (lncRNA) that has never been investigated in cervical cancer (CC). Thus, the potential function and molecular mechanism remain unclear. Our study explored the biological function of *AC010883.5* to determine the underlying mechanisms in CC and provide potential therapeutic targets for improving the clinical treatment strategy. We used quantitative real-time polymerase chain reaction to measure mitochondrial RNA levels and western blot to measure the protein levels of target genes. Further, we used Cell Counting Kit-8 and 5‐Bromo-2'-deoxyuridine incorporation assays to evaluate cell proliferation in vitro. Cell apoptosis was analyzed by flow cytometry. Cell invasion was analyzed by wound healing and Transwell migration assays was ued to analyze cell migration. Finally, the biological function and mechanism of *AC010883.5* in CC growth were evaluated by in vivo xenograft assay. *AC010883.5* was enhanced in CC tissues and cell lines, and enhanced *AC010883.5* expression accelerated CC cell proliferation, migration, and invasion and induced epithelial–mesenchymal transition in vitro and in vivo. *AC010883.*5 also activated the mitogen-activated protein kinase (MAPK) signaling pathway by promoting phosphorylation of extracellular signal-regulated kinase 1/2 (i.e., ERK1/2) and MAPK kinase 1/2 (i.e., MEK1/2). Blocking the MAPK signaling pathway could counteract the pro-proliferative, pro-migrative, and pro-invasive effects of *AC010883.5* over-expression. We found that the lncRNA, AC010883.5, is an oncogenic molecule involved in CC tumor progression via dysregulation of the MAPK signaling pathway, implying that *AC010883.5* could be a tumor progression and therapeutic response biomarker.

**Supplementary Information:**

The online version contains supplementary material available at 10.1186/s12885-023-10825-2.

## Introduction

Cervical cancer (CC) is the second most common malignancy with the highest mortality in females worldwide, ranking just after breast cancer. Globally, there are almost 527 000 new cancer cases and 265 000 patient deaths per year [1]. Despite worldwide screening and advances in early diagnosis and targeted treatments, the overall prognosis of patients with advanced CC remains poor. Therefore, it is imperative to elucidate the pathogenesis underpinning carcinogenesis and progression.

Long non-coding RNAs (lncRNAs) are RNA transcripts longer than 200 nucleotides, often polyadenylated, and devoid of evident open reading frames [2]. Accumulating evidence suggests that lncRNAs participate in CC progression. For example, HOX transcript antisense RNA (i.e., *HOTAIR*) gene expression is strictly controlled by the human papillomavirus (HPV) E7 protein [3], acting as a sponge to alter the miR-143-3p/BCL2 axis favoring cancer cell growth [4] and the miR-129-5p/RPL14 axis promoting cell proliferation and metastasis [5]. Further, miR-145 sponging by metastasis-associated lung adenocarcinoma transcript 1 (i.e., *MALAT1*) was involved in radio-resistance in radiotherapy [6] and identified as a potential therapeutic target and prognostic biomarker [7].

Our research group screened out five lncRNAs most related to the biological functions of cervical cancer through bioinformatics analysis, and determined that *AC010883.5* has the strongest ability to regulate cell growth compared with other lncRNAs through cell experiments. To our knowledge, the lncRNA, homo sapiens chromosome 2 clone RP11-339H12 (*AC010883.5*), located on human chromosome 2p, has never been studied in CC. Our previous research on *AC010883.5* was limited to cell proliferation assays and the correlation between its expression and clinical pathology. Thus, the potential function and molecular mechanism remain unclear. Our study explored the biological function of *AC010883.5* to determine the underlying mechanisms in CC and provide potential therapeutic targets for improving the clinical treatment strategy.

## Materials and methods

### Clinical samples

We collected ten CC and paracancerous tissue pairs from patients with CC from the East Hospital affiliated with the Tongji University of Medicine between 2019 and 2020. Patients pathologically diagnosed with CC with no history of radiochemotherapy or other adjuvant therapies were included. Patients with distant metastasis or other malignant tumors or who were pregnant or lactating were excluded. The Ethics Committee of the Affiliated Tumor Hospital of Xinjiang Medical University approved the present study. All patients have signed the written informed consent forms before surgical resection. Following collection, tissue samples were snap-frozen and stored at − 80 °C before use.

### Cell culture and reagents

CC-derived cell lines (SiHa, Hela, Caski, and C-33A) and a normal epithelial-derived cell line (HcerEpic) were obtained from the Institute of Cell Research, Chinese Academy of Sciences (Shanghai, China) for functional analyses. Hela and SiHa were cultured in Dulbecco’s modified Eagle’s medium (DMEM; Procell, Wuhan, China), Caski in Roswell Park Memorial Institute medium (RPMI; Invitrogen, Carlsbad, USA), and C-33A in minimal essential medium (MEM; Invitrogen, Carlsbad, USA) supplemented with 10% fetal bovine serum (FBS) and 1% penicillin/streptomycin (HyClone, Logan, UT, USA) with 5% carbon dioxide (CO_2_) at 37 °C. When appropriate, the cells were treated with 10 μM of PD98059 (MCE; Beverly, MA, USA).

### AC010883.5 knockdown and overexpression

For in vitro experiments, to knock down *AC010883.5* expression in SiHa cells, small interfering RNAs (siRNAs) targeting AC010883.5 (siRNA1-siRNA4) and a negative control (siNC) were purchased from GenePharma (Shanghai, China). The siRNA sequences were as follows: siRNA1, CUACUACCCUUCCAAAGCU; siRNA2, CUCAAGAUAACUAUUACCA; siRNA3, CAAGACACAUCCUAGAGCA; siRNA4, GAUAACUAUUACCAUCCCA. To upregulate *AC010883.5* expression in C-33A cells, the *AC010883.5* sequence was inserted into pcDNA3.1 vectors to construct AC010883.5 expression vectors (oeAC010883.5). Empty plasmid pcDNA3.1 was used as a negative control. For subsequent experiments, SiHa and C-33A were harvested 24 h after siRNA or plasmid transfection. *AC010883.5* knockdown and overexpression efficiencies were detected by quantitative real-time polymerase chain reaction (qRT-PCR). The two most effective siRNAs were used in the functional experiments.

For in vivo experiments, short hairpin RNA (shRNA) targeting *AC010883.5* generated from siRNA2 and a scrambled shRNA were cloned into pLKO.1-puro (Addgen, MA, USA). Lentiviral particles were generated in 293 T cells by co-transfecting a lentiviral plasmid and packaging plasmid (psPAX and pMD2.G, Addgene, MA, USA) using Lipofectamine 2000 (Thermo Fisher Scientific, NY, USA). After 48 h, the transfected lentiviral supernatants were filtered for further infection of SiHa cells and prepared for tumor formation in a nude mice experiment.

### Cell proliferation assay

The proliferative capacity was evaluated by Cell Counting Kit-8 (CCK-8) assay (SAB, MA, USA, CP002) at 0, 24, 48, and 72 h. Cells (3 × 10^4^/well) were seeded into 96-well plates and incubated at 37 °C in 5% CO_2_ and treated with CCK-8 every 24 h. The absorbance was measured at 450 nm, and a proliferation curve was plotted based on optical density values detected by a microplate reader (Perlove Medical; DNM-9602).

A 5‐Bromo-2'-deoxyuridine (BrdU) incorporation assay was performed with the Cell-Light™ BrdU Apollo®643 In Vitro Imaging Kit (RiboBio, Guangzhou, China). Cells (5 × 10^4^/well) were harvested and treated with BrdU, fixed for 30 min and incubated using peroxidase-coupled anti-BrdU-antibody (Sigma Aldrich) for 1 h. The samples were analyzed using a Zeiss fluorescence photomicroscope (Carl Zeiss, Oberkochen, Germany) by randomly counting ten fields.

### qRT-PCR

The gene mRNA expression levels were estimated by qRT-PCR. Total RNA was extracted using TRIzol reagent (1596–026, Invitrogen, Carlsbad, USA). First-strand complementary DNA was synthesized from total RNA using the SuperScript II first-strand synthesis system (Invitrogen Carlsbad, USA). The target gene primer sequences were: glyceraldehyde 3-phosphate dehydrogenase (*GAPDH*): F 5′-TGCACCACCAACTGCTTAGC-3′, R 5′-GGCATGGACTGTGGTCATGA-3′; *AC010883.5*: F 5’-AAGGCAAACTCTGCAAAGACAG-3’, R 5′-TCCAAGGAAAGGGCAAACC-3’. The relative mRNA levels were quantified using the 2^−ΔΔCT^ method with *GAPDH* as an internal control.

### Cell apoptosis

Annexin V and propidium iodide (PI) staining were applied using an Annexin V-FITC Apoptosis Detection kit (Beyotime Biotechnology, Shanghai, CHN, C1062). Cells (3 × 10^4^/well) were harvested and resuspended in staining buffer then stained with 10 μL of Annexin V and 5 μL of PI solution in the dark for 15 min. Apoptotic cells were analyzed via a BD FACSCanto™ II flow cytometer (BD Biosciences, USA), after which FlowJo Software (v10.4; FlowJo LLC, Ashland, OR, USA) was used for data analysis.

### Wound-healing assay

For the wound healing assay, 8 × 10^5^ cells were seeded into 6—well plates until reaching to a density of 60%. Cells were wounded using a pipette tip and incubated in serum-free medium. Cell images were captured at 12-h intervals and photographed with an inverted microscope. Then, the wound closure percentage was measured. Scratch areas were measured using the Image J software. Wound healing percentage (%) was quantified using the percentage change in the normalized measurement area divided by the original open area according to the formula: Wound healing percentage (%) = [A (0)—A (t)/A (0)] × 100 where the area (A) at time zero (0) and the area after incubation time (t) were used to calculate the percent wound healing [8].

### Transwell assay

For the migration assay, cells were suspended in a serum-free medium, then 5 × 10^4^ cells in 200 μL of serum-free medium were seeded into the upper chamber coated with or without Matrigel. The lower chamber received 700 μL of complete medium supplemented with 10% FBS. After 24 h, the invaded or migrated cells were fixed with 50% methanol, then stained with 0.5% crystal violet solution for 30 min at room temperature. The numbers of invaded and migrated cells were evaluated via microscopy (Olympus Corporation) by randomly selecting three fields.

### Western blotting

Radio-Immunoprecipitation Assay (i.e., RIPA) buffer (Beyotime Biotechnology, CA, China) supplemented with 1 nM of benzylsulfinyl fluoride was added to extract total proteins from the cells or tumor samples, followed by a bicinchoninic acid (i.e., BCA) assay (Beyotime) to quantify the protein concentrations. The proteins (30 μg) were separated using a 10% sodium dodecyl sulfate–polyacrylamide gel electrophoresis (i.e., SDS-PAGE) gel, then transferred to polyvinylidene fluoride membrane (EMD Millipore, Burlington, MA, USA). The membrane was blocked for 2 h with non-fat milk followed by primary antibody incubation, including E-cadherin (1:1000; Abcam; No. ab231303), N-cadherin (1:1000; Abcam; No. ab18203), ERK1/2 (1:1000; Abcam; No. ab184699), p-ERK1/2 (1:1000; Abcam; No. ab223500), MEK1/2 (1:500; Affinity; No. AF6385), p-MEK1/2 (1:500; Affinity; No. AF8035), and GAPDH (1:1000; Proteintech; #60,004–1-1G) at 4 °C overnight. The blots were washed with TBS-T and treated with horseradish peroxidase-conjugated polyclonal goat anti-rabbit immunoglobulin G (IgG; 1:1000; No. A0208) or polyclonal goat anti-mouse IgG (1:1000; Beyotime; No. A0216) for 1 h at ambient temperatures. An enhanced chemiluminescence kit (Beyotime; WBKLS0100) was used to assess the protein bands.

### Mouse tumor xenograft model

Female BALB/c nude mice (5–6 weeks old, 15–20 g) from Shanghai Slark Experimental Animal Company were housed in specific pathogen-free conditions. Animals were randomized into two groups (six mice per group). SiHa cells were suspended in phosphate-buffered saline at 2 × 10^7^/mL, then 100 μL were subcutaneously injected to the right flank of each mouse. The tumor volume was measured every three days and calculated as follows: tumor volume [mm^3^] = length × width [2] × 0.5. Mice were sacrificed 33 days after implantation, and the specimen was collected for weight measurements and in vitro assays. All experiments involving animals were approved by the Animal Care and Use Committee at Tongji University and followed by Guidelines for the ethical review of laboratory animal welfare People’s Republic of China National Standard GB/T 35,892–2018.

### Statistical analyses

All data analysis was performed using GraphPad Prism version 6.0 (GraphPad Software, La Jolla, USA) and SPSS version 22 (IBM Corp., Armonk, NY, USA). The data were expressed as means ± standard deviations. Continuous data were analyzed using Student’s t-test or one-way analysis of variance. *p* < *0.05* was considered statistically significant.

## Results

### *AC010883.5* expression in CC tissues and cell lines

**Fig. 1:**
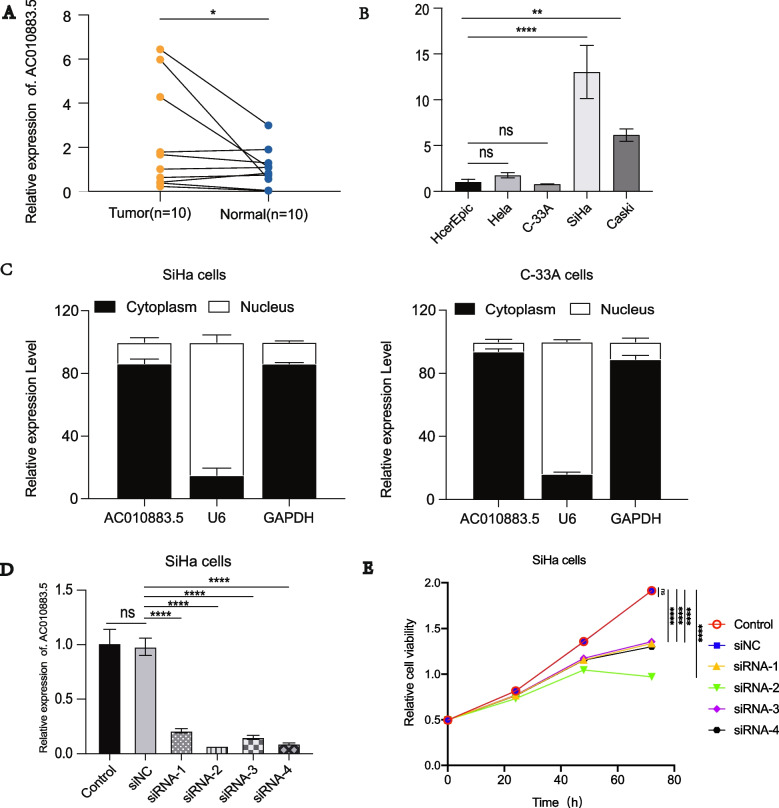
The relative expression and subcellular location of *AC010883.5*. **A**. qRT-PCR analysis of *AC010883.5* expression in CC and adjacent cervical tissues from ten patients. **B**. qRT-PCR analyses of *AC010883.5* expression in four CC cell lines and one normal cell line. **C**. An investigation of the subcellular location of *AC010883.5* in SiHa and C-33A cells by qPCR. The original magnification was 400 × . **D**. *AC010883.5* expression in SiHa cells transfected with si-NC or one of four experimental siRNAs. **E**. CCK-8 analysis of AC010883.5 expression in SiHa cells transfected with si-NC or siRNA 1 to 4. The error bars represent the mean ± SD of at least three experiments. *N* = 3. All qPCR results were normalized to *GAPDH*. *P < 0.05, **P < 0.01, and ***P < 0.001. Abbreviations: qRT-PCR, quantitative real-time polymerase chain reaction; CC, cervical cancer; si-NC, negative control siRNA; siRNA, small interfering RNA; CCK-8, Cell Counting Kit‐8; DAPI, 4′,6‐diamidino‐2‐phenylindole

*AC010883.5* expression was significantly enhanced in the cancer tissues compared with the adjacent tissues (Fig. 1A). Generally, *AC010883.5* was expressed more in the CC cell lines than in the HcerEpic cell line (Fig. 1B). The function of lncRNAs is strongly linked to subcellular distribution in cells. Therefore, qRT-PCR was performed to distinguish the subcellular distribution of *AC010883.5* in the SiHa and C-33A cell lines using *GAPDH* and *U6* as cytoplasmic and nuclear references, respectively. *AC010883.5* was substantially enriched in the cytoplasm (Fig. 1C). Further, *AC010883.5* knockdown with siRNAs (siAC010883.5–1, siAC010883.5–2, siAC010883.5–3, siAC010883.5–4) dramatically decreased *AC010883.5* expression in SiHa cells (Fig. 1D). The CCK-8 analysis also demonstrated that viable SiHa cells were remarkably decreased after silencing *AC010883.5* (Fig. 1E).

### Silenced *AC010883.5* suppressed SiHa cell proliferation, induced apoptosis, and repressed cell migration and invasion

**Fig. 2:**
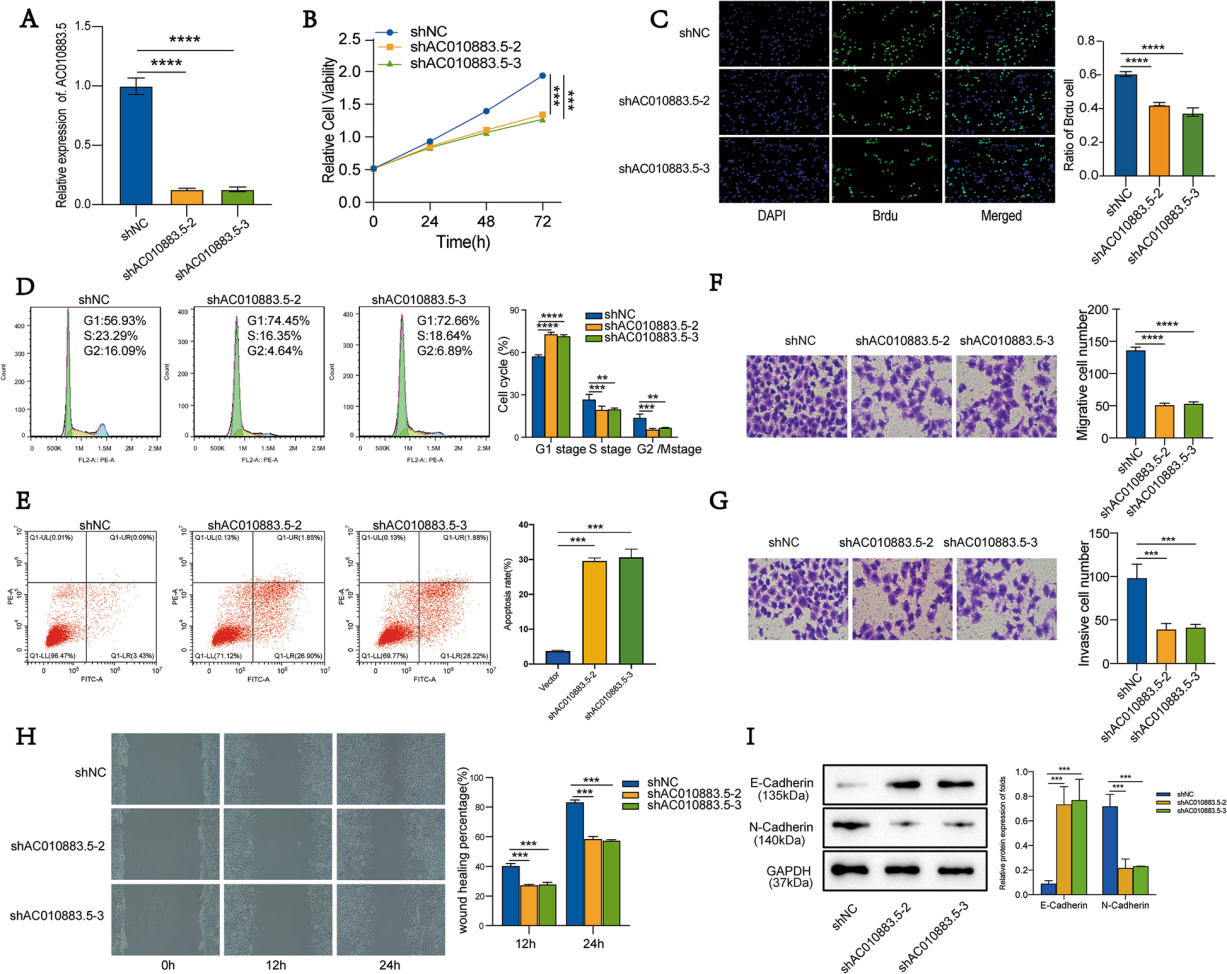
Silencing *AC010883.5* suppressed proliferation and progression in SiHa cells. **A**. qRT-PCR analysis of *AC010883.5* expression in SiHa cells transfected with shRNA-2 and shRNA-3. **B**. CCK-8 analysis of viable SiHa cells under AC010883.5 treatment (silenced vs. control). **C**. BrdU incorporation analysis of proliferative SiHa cells under AC010883.5 treatment (silenced vs. control). **D**. Cell cycle quantification of SiHa cells using flow cytometry. **E**. Flow cytometry analysis of apoptotic SiHa cells under AC010883.5 treatment (silenced vs. control). **F**. Transwell migration analysis of migrative SiHa cells under AC010883.5 treatment (silenced vs. control). **G**. Transwell invasion analysis of invasive SiHa cells under AC010883.5 treatment (silenced vs. control); **H**. Wound healing analysis of migrative SiHa cells under AC010883.5 treatment (silenced vs. control). **I**. Western blots of E-cadherin and N-cadherin in SiHa cells under AC010883.5 treatment (silenced vs. control). *N* = 3; Abbreviations: qRT-PCR, quantitative real-time polymerase chain reaction; shRNA, short hairpin RNA; CCK-8, Cell Counting Kit‐8; BrdU, 5‐Bromo-2'-deoxyuridine. *P < 0.05, **P < 0.01, and ***P < 0.001

To discriminate the effects of *AC010883.5* on cervical cells, we first generated cells with stably knocked down *AC010883.5* (transfection with siAC010883.5–2 and siAC010883.5–3) and SiHa control cells (transfection with siNC, an empty control); we evaluated the efficiency by qRT-PCR (Fig. 2A).

In SiHa cells, silencing *AC010883.5* suppressed cell survival (CCK-8 assay; Fig. 2B) and significantly restrained cell proliferation (BrdU incorporation assay; Fig. 2C). Silencing *AC010883.5* also arrested the cell cycle at the G1 phase (Fig. 2D) and induced apoptosis (flow cytometry, Fig. 2E). Furthermore, silencing *AC010883.5* greatly inhibited cells migration (Transwell migration and wound healing assays; Fig. 2F, H) and remarkably slowed cell invasion (Transwell invasion assay; Fig. 2G).

Epithelial–mesenchymal transition (EMT) is one of the most important indicators of cancer metastasis. Thus, we explored the correlation between *AC010883.5* and EMT. Silencing *AC010883.5* decreased the N-cadherin protein level and enhanced the E-cadherin protein level in SiHa cells compared to control cells (Fig. 2I), indicating that *AC010883.5* could be involved in EMT.

### Overexpressing *AC010883.5* accelerated C-33A cell proliferation, inhibited apoptosis, and promoted cell migration and invasion

**Fig. 3:**
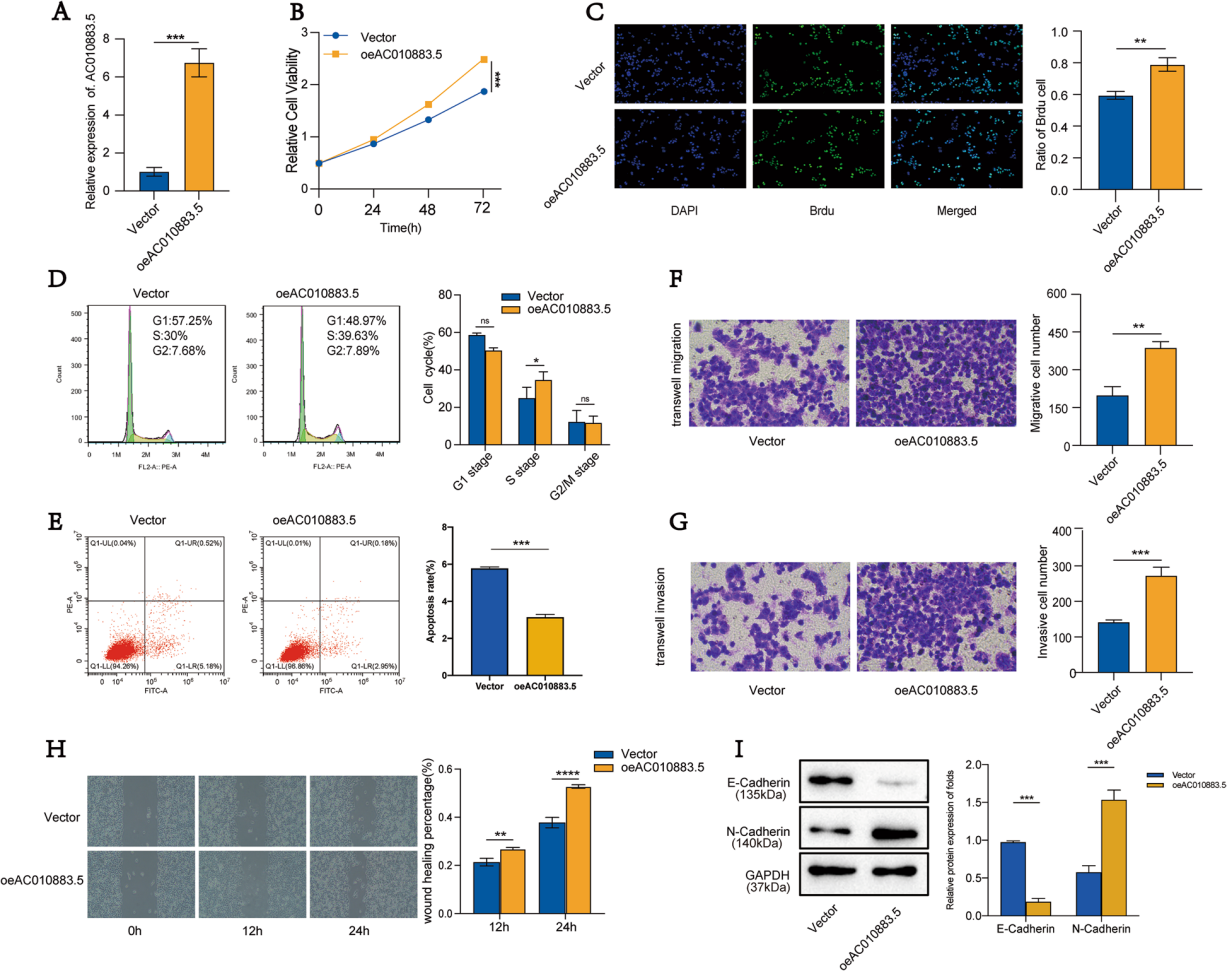
Overexpressed *AC010883.5* promoted proliferation and progression in C-33A cells. **A**. qRT-PCR analyses of *AC010883.5* expression in C-33A cells transfected with the oeAC010883.5 vector. **B**. CCK-8 analysis of viable C-33A cells under AC010883.5 treatment (overexpressed vs. control). **C**. BrdU incorporation analysis of proliferative C-33A cells under AC010883.5 treatment (overexpressed vs. control). **D**. Quantification of flow cytometry results in C33-A cells under AC010883.5 treatment (overexpressed vs. control). **E**. Flow cytometry analysis of apoptotic C-33A cells under AC010883.5 treatment (overexpressed vs. control). **F**. Transwell migration analysis of migrative C-33A cells under AC010883.5 treatment (overexpressed vs. control). **G**. Transwell invasion analysis of invasive C-33A cells under AC010883.5 treatment (overexpressed vs. control). **H**. Wound healing analysis of migrative C-33A cells under AC010883.5 treatment (overexpressed vs. control). **I**. Western blots of E-cadherin and N-cadherin in C-33A cells under AC010883.5 treatment (overexpressed vs. control). Abbreviations: qRT-PCR, quantitative real-time polymerase chain reaction; CCK-8, Cell Counting Kit‐8; BrdU, 5‐Bromo-2'-deoxyuridine

We also constructed stably overexpressed *AC010883.5* cells (transfection with an AC010883.5 overexpression vector) and control C-33A cells (transfection with an empty vector); we evaluated the efficiency by qRT-PCR (Fig. 3A).

In C-33A cells, overexpressing *AC010883.5* promoted cell survival (CCK-8 assay; Fig. 3B) and significantly improved cell proliferation (BrdU incorporation assay; Fig. 3C). Overexpressing *AC010883.5* also induced cell cycle transitions; the ratio of cells in the G1 phase decreased while the ratio in the S phase increased (Fig. 3D). Overexpressed *AC010883.5* repressed cell apoptosis (flow cytometry, Fig. 3E), increased cell migration (wound healing assay; Fig. 3F, H), and markedly accelerated cell invasion (Transwell migration assay; Fig. 3G). Regarding the EMT analysis, overexpressing *AC010883.5* enhanced the N-cadherin protein level and decreased the E-cadherin protein level in C-33A cells (Fig. 3I).

### *AC010883.5* activates the mitogen-activated protein kinase (MAPK) signaling pathway

**Fig. 4:**
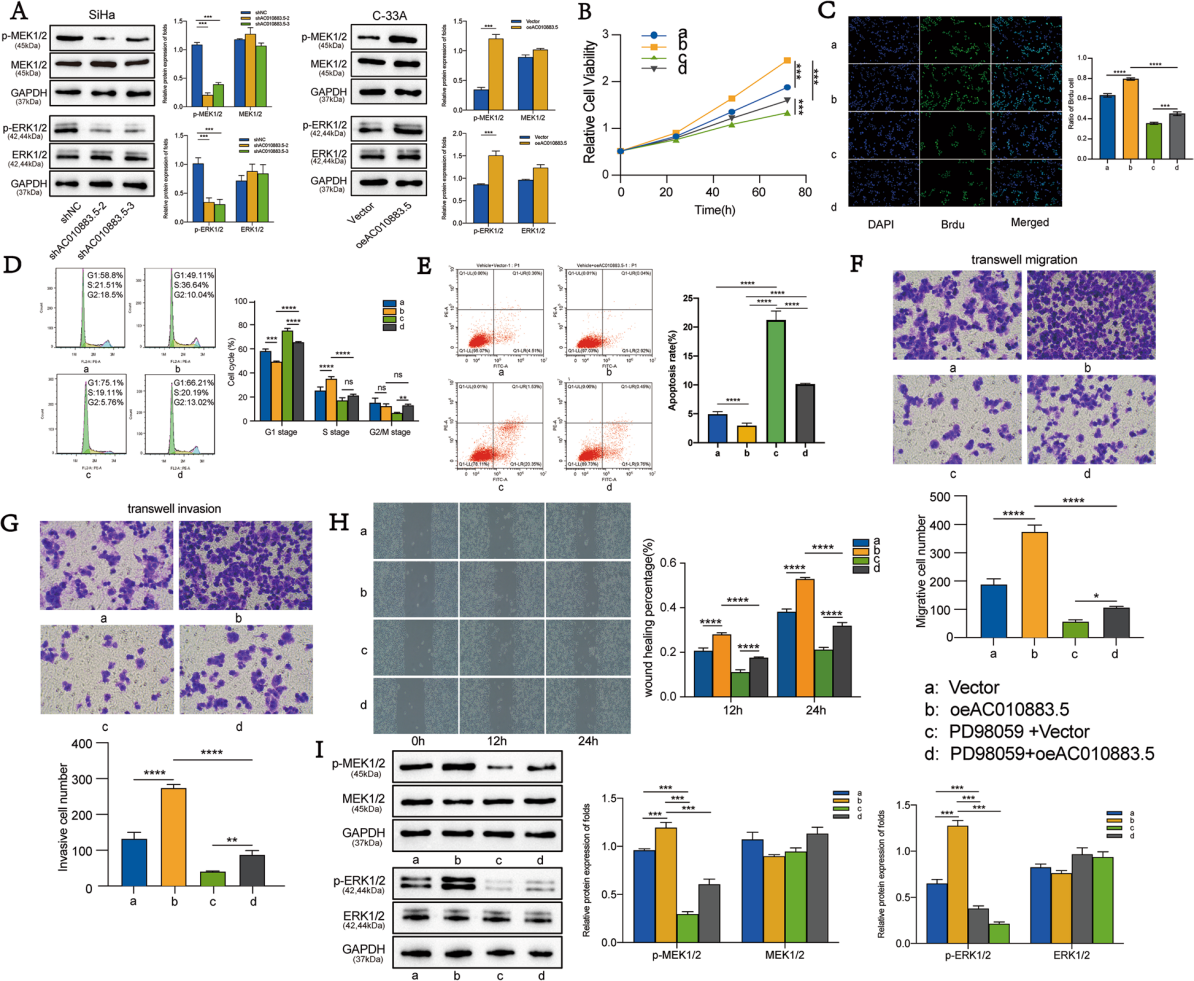
Overexpressed *AC010883.5* activates the MAPK signaling pathway and promoted C33A cell progression. **A**. Western blots of ERK1/2, p-ERK1/2, MEK1/2, and p-MEK 1/2 protein in SiHa cells under AC010883.5 treatment (silenced vs. control) and C-33A cells under AC010883.5 (overexpressed vs. control). **B**. CCK-8 analysis of viable C-33A cells in a vehicle or PD98059-treated C-33A cells under AC010883.5 treatment (overexpressed vs. control). **C**. BrdU incorporation analysis of proliferative C-33A cells in a vehicle or PD98059-treated C-33A cells under AC010883.5 treatment (overexpressed vs. control). **D**. Quantification of flow cytometry results in C33-A cells under AC010883.5 treatment (overexpressed vs. control). **E**. Flow cytometry analysis apoptotic C-33A cell in a vehicle or PD98059-treated C-33A cells under AC010883.5 treatment (overexpressed vs. control). **F**. Transwell migration analysis of migrative C-33A cell in a vehicle or PD98059-treated C-33A cells under AC010883.5 treatment (overexpressed vs. control). **G**. Transwell invasive analysis of invasive C-33A cell in a vehicle or PD98059-treated C-33A cells under AC010883.5 treatment (overexpressed vs. control). **H**. Wound healing analysis of migrative C-33A cells in vehicle or PD98059-treated C-33A cells in a vehicle or PD98059-treated C-33A cells under AC010883.5 treatment (overexpressed vs. control). **I**. Western blots of MAPK signal molecules in a vehicle or PD98059-treated C-33A cells under AC010883.5 treatment (overexpressed vs. control). *N* = 3; *P < 0.05, **P < 0.01, and ***P < 0.001. a: Vector; b: oeAC010883.5; c: PD98059 + Vector; d: PD98059 + oeAC010883.5; Abbreviations: MAPK, mitogen-activated protein kinase; ERK1/2, extracellular signal-regulated kinase 1/2; p-ERK1/2, phosphorylated ERK1/2; MEK1/2, MAPK kinase 1/2; p-MEK1/2, phosphorylated MEK1/2; CCK-8, Cell Counting Kit‐8; BrdU, 5‐Bromo-2'-deoxyuridine

The MAPK signaling pathway is of known importance in CC progression [9]. Therefore, we investigated the role of this pathway in cell phenotype adjustments under the influence of *AC010883.5*. We evaluated the phosphorylation levels of MAPK kinase (MEK; p-MEK) and extracellular signal-regulated kinase (ERK) 1/2 (p-ERK1/2), key biomarkers of MAPK signaling pathway activation, by western blot. p-MEK1/2 and p-ERK1/2 significantly increased in *AC010883.5* overexpressing cells and decreased in *AC010883.5* knockdown cells (Fig. 4A). Therefore, we hypothesized that the MAPK pathway was involved in *AC010883.5* function in CC cells.

The MAPK pathway inhibitor, PD98059, was used to block the activation signal in *AC010883.5* overexpressed C-33A cells to investigate whether AC010883.5 promotes CC progression through the MAPK signaling pathway. Proliferation and cell cycle analyses revealed that PD98059 suppressed *AC010883.5*-mediated cell proliferation and cell cycle transition and reversed apoptosis inhibition in C-33A cells (Fig. 4B–E). Consistently, PD98059 suppressed *AC010883.5*-mediated cell migration and invasion in C-33A cells (wound healing and Transwell migration assays; Fig. 4F–H). Moreover, PD98059 reversed the effects of *AC010883.5* overexpression on the ERK1/2 and MEK1/2 phosphorylation (western blot; Fig. 4I). These results suggest that *AC010883.5* promotes proliferation, invasion, and migration of CC cells by activating the MAPK pathway.

### *AC010883.5* knockdown inhibited CC tumor growth in vivo

**Fig. 5:**
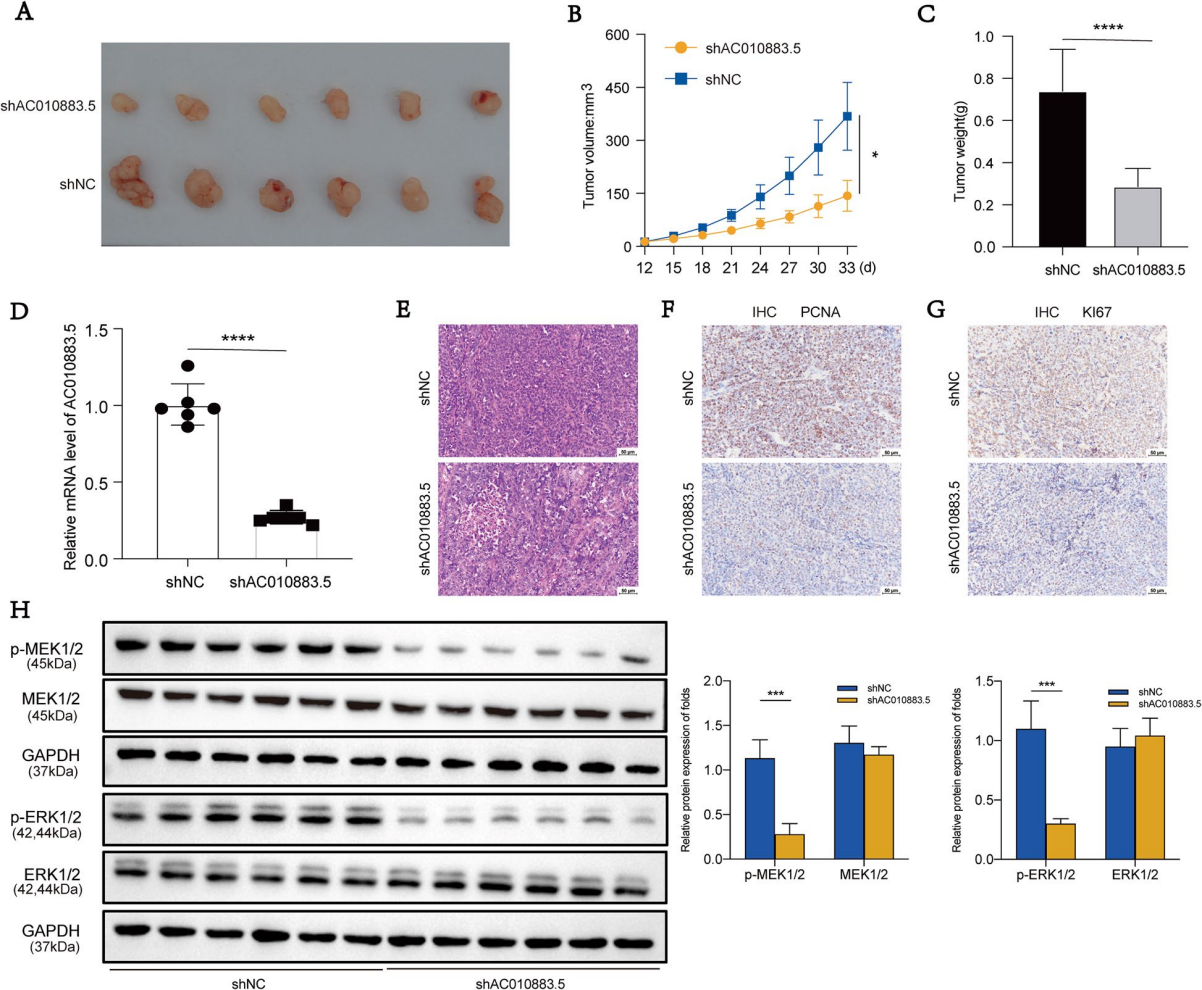
Silencing *AC010883.5* inhibited tumor cell proliferation and progression by inactivating the MAPK signaling pathway. **A**. Tumor xenografts images on the day of sacrifice (day 33; *N* = 6). **B**. Dissected tumor tissue growth with AC010883.5 pLVX-shRNAs-puro SiHa cells. **C**. Dissected tumor tissue weight with AC010883.5 pLVX-shRNAs-puro SiHa cells. **D**. *AC010883.5* expression in SiHa cells transfected with pLVX-shRNAs-puro in tumor tissue. **E**–**G**. *PCNA* and *Ki67* expression in C-33A cells were detected by immunohistochemistry staining of the tumor tissue. H. ERK1/2, p-ERK, MEK1/2, and p-MEK protein levels with pLVX-shRNAs-puro SiHa cells by western blot; *N* = 6; *P < 0.05, **P < 0.01, and ***P < 0.001. Abbreviations: MAPK, mitogen-activated protein kinase; PCNA, proliferating cell nuclear antigen; ERK1/2, extracellular signal-regulated kinase 1/2; p-ERK1/2, phosphorylated ERK1/2; MEK1/2, MAPK kinase 1/2; p-MEK1/2, phosphorylated MEK1/2

To explore the function of *AC010883.5* in vivo, nude mice were used to establish a xenograft mouse model by subcutaneously injecting control or stably knocking down *AC010883.5* SiHa cells. The tumor sizes and weights of the shAC010883.5 mice were significantly less than the control mice (Fig. 5A–C). As expected, the shAC010883.5 mice had lower *AC010883.5* expression than the control mice (Fig. 5D).

Next, we evaluated proliferation biomarkers in the xenograft tumors. Proliferating cell nuclear antigen (*PCNA*) and *Ki67* expression were significantly down-regulated in the shAC010883.5 mice compared with the control mice (Fig. 5E-G), indicating that *AC010883.5* downregulation suppressed tumor growth in vivo, consistent with in vitro.

More importantly, *AC010883.5* knockdown downregulated the ERK1/2 and MEK1/2 phosphorylation levels in xenograft tumors (Fig. 5H). Collectively, these data demonstrate that *AC010883.5* modulates CC growth via the MAPK signaling pathway.

## Discussion

CC is a lethal malignancy posing a serious threat to the lives of females [10]. Despite advances in treatment and screening, the resistance to therapy and high recurrence rates pose challenges to physicians and patients [11, 12]. Therefore, finding new biomarkers to CC improve treatment strategies and prognosis predictions is crucial.

In recent decades, the biological functions of lncRNAs have been widely investigated [13, 14]. Abnormal expression of lincRNAs are considered to be key factors in the tumorigenesis of cancers [15, 16]. Additionally, an increasing number of studies implicate lncRNAs in CC. Several lncRNAs, including *HOTAIR* [17], *H19* [18], mucosa-associated lymphoid tissue 1 (i.e., *MALT1*) [19], growth arrest-specific 5 (i.e., *GAS5*) [20], cervical carcinoma high expressed 1 (i.e., *CCHE1*) [17], and Pvt1 oncogene (i.e., *PVT1*) [21], are crucial in tumorigenesis, invasion, metastasis, and radio-resistance [22–24]. HOTAIR is significantly associated with CC progression, increasing proliferation, migration, invasion, and EMT by increasing enhancer of zeste homolog 2 (i.e., EZH2) and trimethylation of histone H3 at lysine 27 (i.e., H3K27me3) binding of the antagonist naked cuticle homolog 1 (i.e., *NKD1*) [3]. Differentiation antagonizing non-protein coding RNA (i.e., DANCR) has also been significantly implicated in CC development, augmenting cell adhesion, viability, invasiveness, and migration by down-regulating *p‐GSK3β* and *β‐catenin* expression and suppressing Wnt/β‐catenin pathway activation [25]. Further, H19, controlled by the HPV16 E6 protein, remarkably correlated with CC progression by increasing cell migration, invasion, and proliferation through working as a molecular sponge for miR-138-5p in epithelial cells [26]. These results highlight that lncRNA deregulation occurs in carcinogenesis and progression and exerts both oncogenic and tumor inhibition functions related to CC. *AC010883.5* is located at chromosome 2:43.46, is 216626 bp long [27], and is involved in inflammatory bowel disease [28]. In this study, we reported the function and localization of *AC010883.5* in cells for the first time. Our study showed that *AC010883.5* expression was upregulated in CC tissues and cell lines. We examined the expression of *AC010883.5* in 4 different cervical cancer cell lines, all of which were elevated compared with cervical epithelial cells (HcerEpic). However, the level of *AC010883.5* was higher in SiHa and Caski cells than in HeLa and C33A, which was caused that cell lines derived from different individuals have different genetic backgrounds. In addition, we discovered that *AC010883.5* was substantially enriched in the cytoplasm. *AC010883.5* depletion suppressed CC cell proliferation, migration, invasion, and epithelial-to-mesenchymal transition (EMT).

Furthermore, we explored the molecular mechanisms by which knockdown of *AC010883.5* inhibited CC cell growth in vitro. The MAPK signaling pathway modulates the cell’s response to proliferation, migration, apoptosis, and stress. Further, *ERK1/2* and *MEK1/2* expression and hyperactivation play an essential role in the survival and development of tumor cell proliferation, migration, invasion, and EMT [29, 30]. An aberrant MAPK signaling pathway is a critical regulator of carcinogenesis and the development of multiple tumor types [31]. It has been recognized that lncRNAs can influence cancer occurrence and development via the MAPK pathway [32, 33]. Liao et al. discovered that *lncRNA-CCHE1* promoted the proliferation, migration, and invasion in non-small cell lung cancer (NSCLC) cell line through the ERK/MAPK pathway [34]. Zhang et al. found that *GINS2* also inhibits cell activity by interfering with the MAPK/ERK pathway and induces cell cycle arrest, thus promoting apoptosis of pancreatic cancer cells [35]. Moreover, Du et al. demonstrated that inhibition of *SLC25A22* by the MAPK/ERK pathway could promote mitochondrial apoptosis, thus inhibiting the growth and proliferation gallbladder carcinoma cells [36]. Therefore, we examined the effects of *AC010883.5* knockdown on the ERK/MAPK pathway. In the present study, we demonstrated that upregulation of *AC010883.5* promote the growth and invasion of tumor by activating this signaling cascade. It was observed that overexpression of *AC010883.5* significantly increased the expression of phosphorylated ERK1/2. However, no detectable changes in the expression of total ERK1/2 protein were observed. To further verify the role of signaling activation and inactivation in *AC010883.5*-aberrant expressed CC, the addition of PD98059 was used to pretreat cells. Results showed that inhibition of MAPK signaling suppressed *AC010883.5* high expression-induced levels of phosphorylated ERK1/2 and MEK1/2. Thus, we concluded that AC010883.5 promoted cell proliferation, invasion, migration, and epithelial-to-mesenchymal transition in cervical cancer by activating the MAPK signaling pathway.

This study had several limitations, including the low number of clinical samples, the lack of a reliable clinical characteristics analysis, and insufficient research on the regulatory mechanism. These limitations might contribute to unilateral results. Future research should include a larger sample size and involve more complex regulation processing to validate our results.

## Conclusions

In summary, in this study, we first found that *AC010883.5* is markedly enhanced in CC tissues and cell lines. Further, it was involved in cell proliferation, invasiveness, and migration and reduced apoptosis, accelerating tumor growth and EMT in vitro and in vivo. We also found that enhanced *AC010883.5* expression induced an ERK1/2 and MEK1/2 phosphorylation increase and activated the MAPK signaling pathway to promote tumor progression. The results demonstrate that *AC010883.5* is a possible CC oncogene, and therapies targeting *AC010883.5* may be of interest.

## Supplementary Information

**Additional file 1.**

## Data Availability

The datasets used and/or analyzed during the current study are available from the corresponding author on reasonable request.
