# Supplementary material for: AC010883.5 promotes cell proliferation, invasion, migration, and epithelial-to-mesenchymal transition in cervical cancer by modulating the MAPK signaling pathway
Source: BMC Cancer. 2023 Apr 21;23:364. doi: 10.1186/s12885-023-10825-2 (PMC10120252; doi:10.1186/s12885-023-10825-2)
Supplement: Supplementary file 1 — Additional file 1. [file 12885_2023_10825_MOESM1_ESM.pdf]

AC010883.5 promotes cell proliferation, invasion, migration, and epithelial-to-mesenchymal transition in cervical cancer by modulating the MAPK signaling pathway

Original data for WB. Blots surrounded by the black border were corresponding to the Figures in the same order and locations. The expression of other proteins which had different molecular weight when conducting the WB ECL-HRP detection process should be detected for other researching purpose. Thus, to avoid acquiring multiple protein bands in one field, which would interfere the results presentation of this study, protein bands which considered to be interest protein were cut prior to hybridisation with antibodies according to the hint of protein marker.

The original blots shown in the revised Figure 2I were listed as follow:

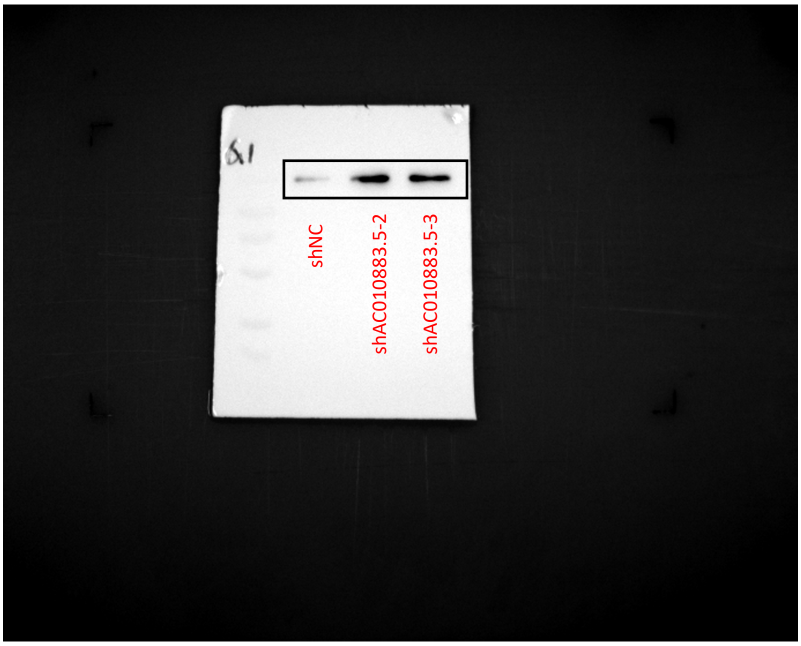

E-cadherin

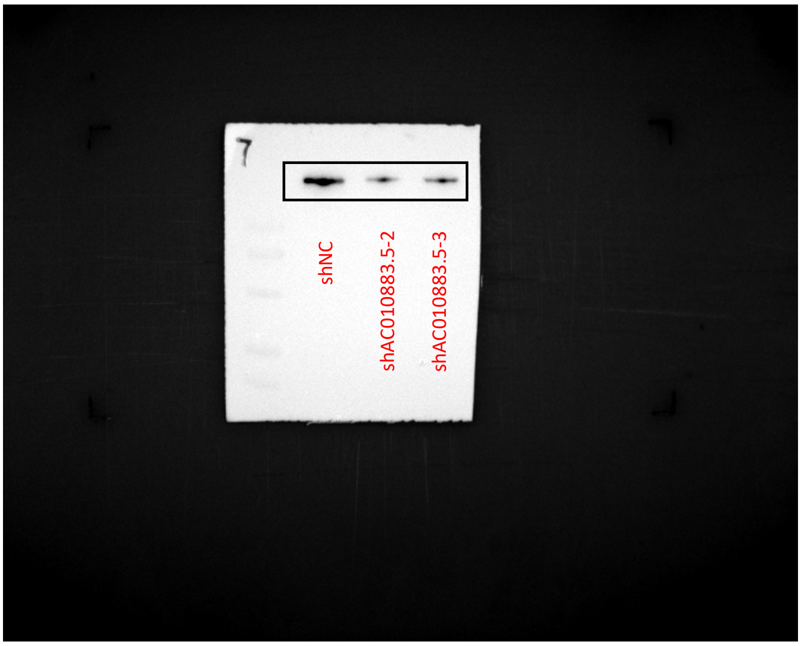

N-cadherin

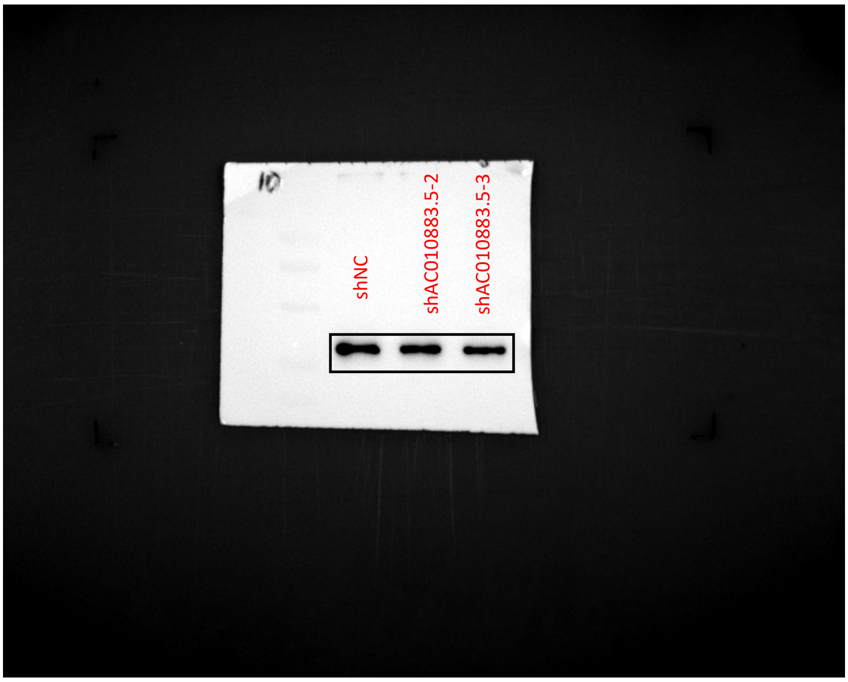

GAPDH

The black boxes show the cropped edge.

The original blots shown in the revised Figure 3I were listed as follow:

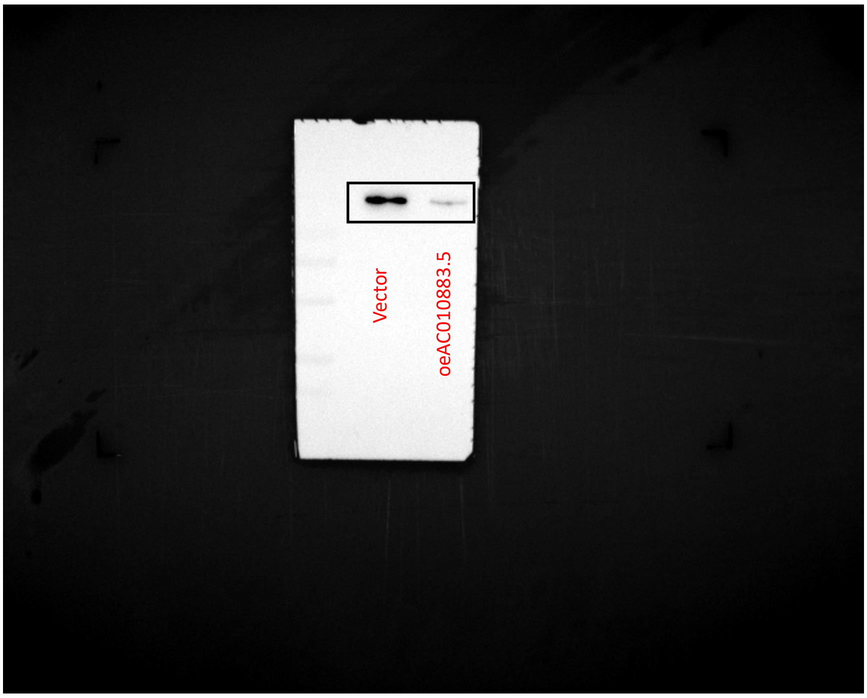

E-cadherin

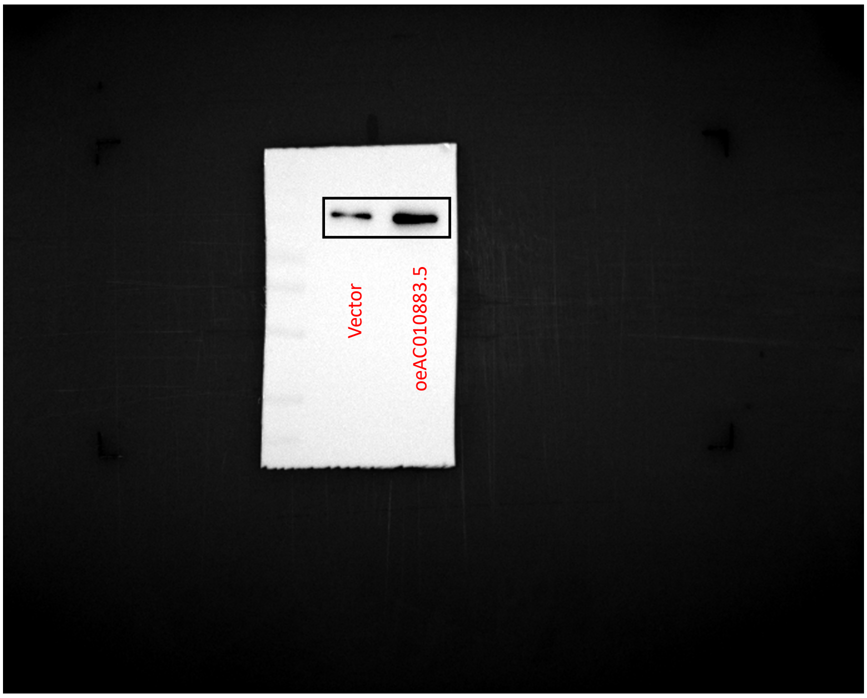

N-cadherin

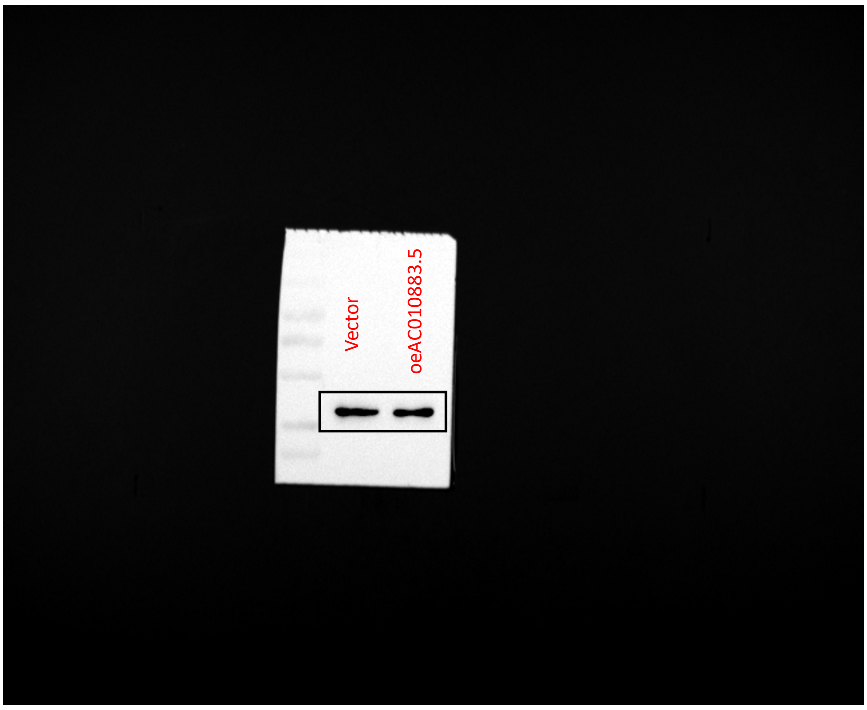

GAPDH

The black boxes show the cropped edge.

The original blots shown in the revised Figure 4A were listed as follow:

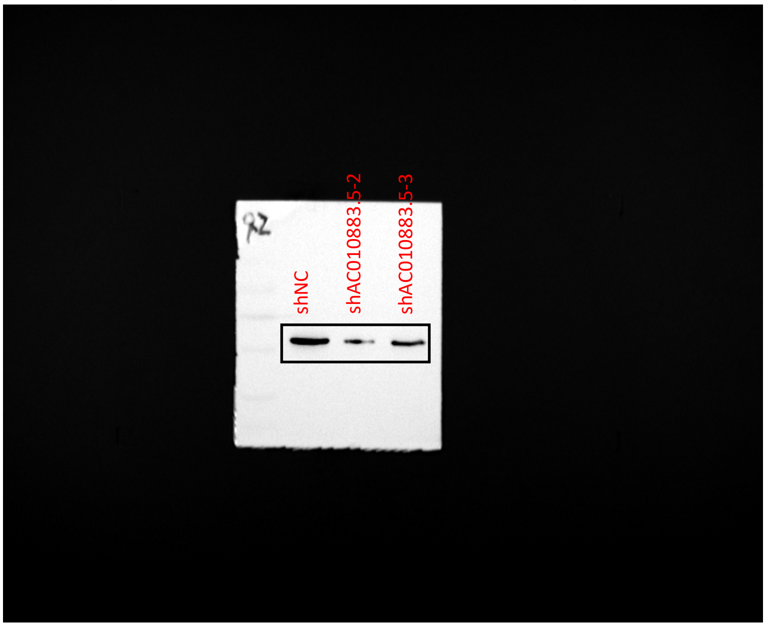

p-MEK1/2

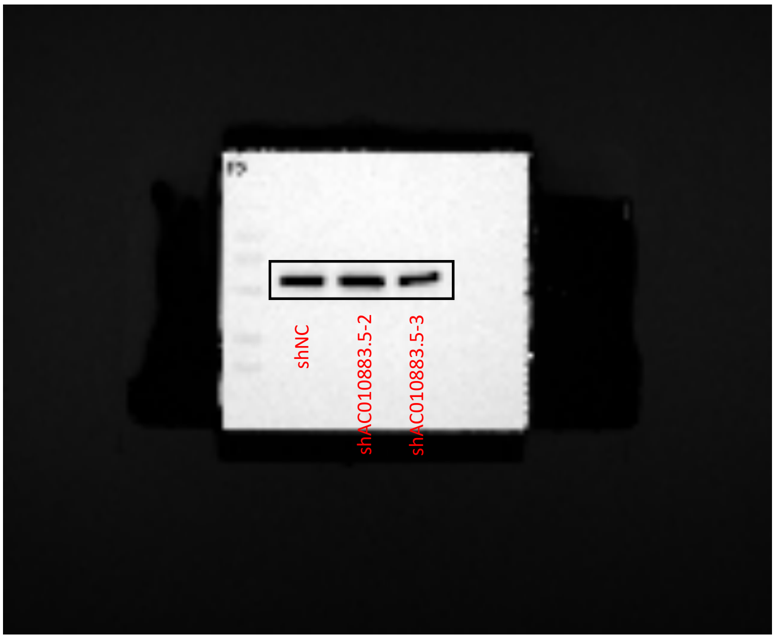

MEK1/2

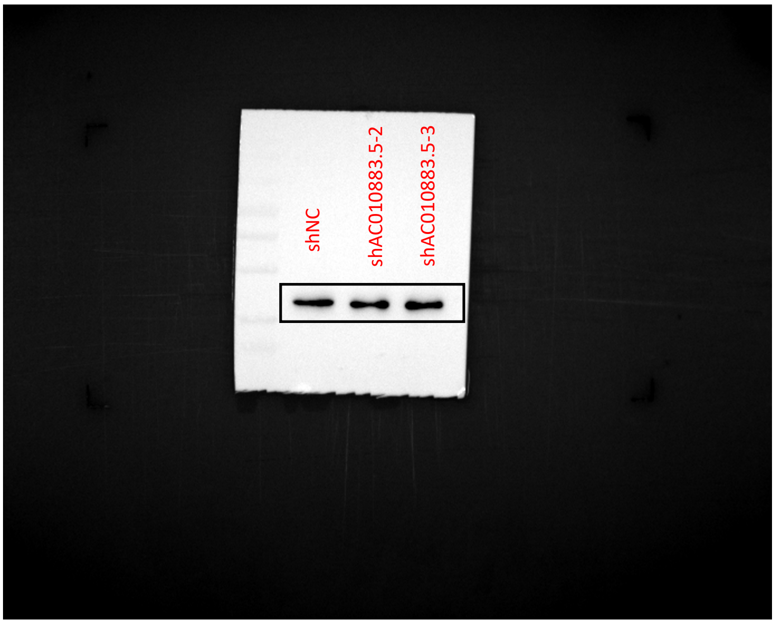

GAPDH

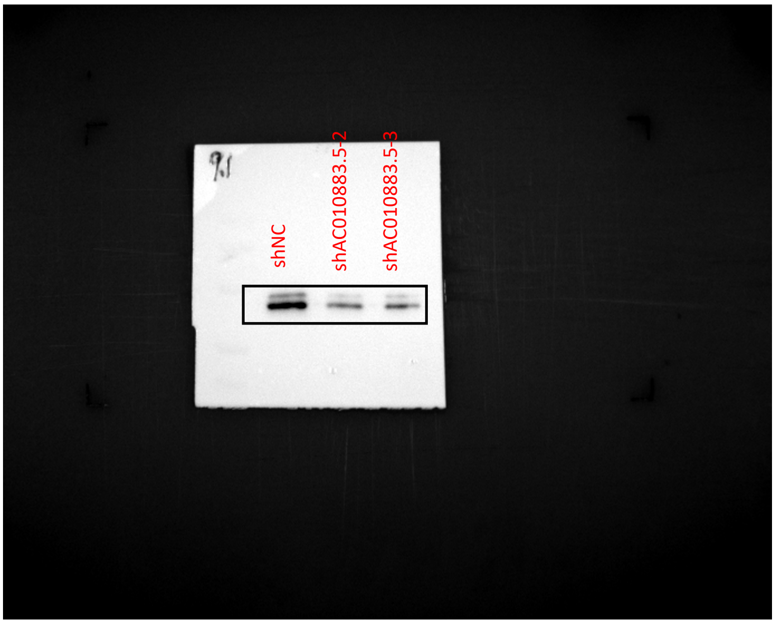

p-ERK1/2

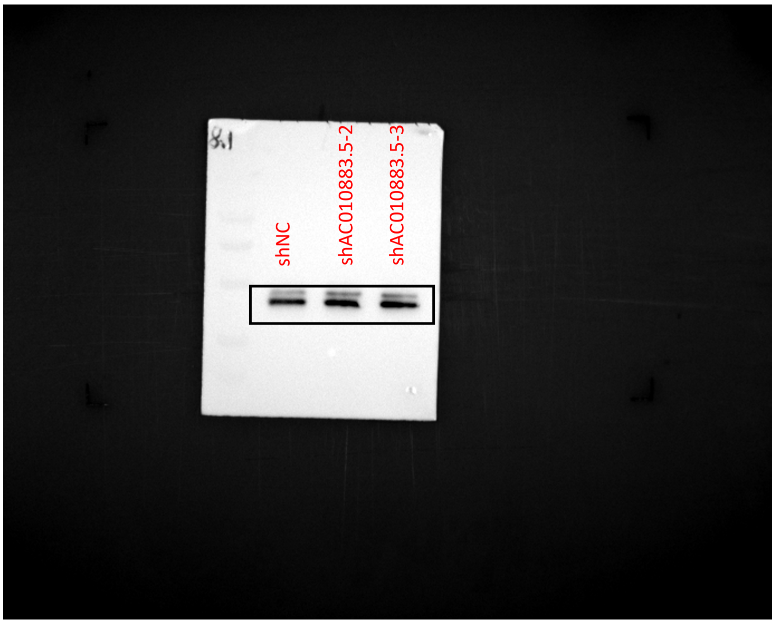
shNC
shAC010883.5-2
shAC010883.5-3

ERK1/2

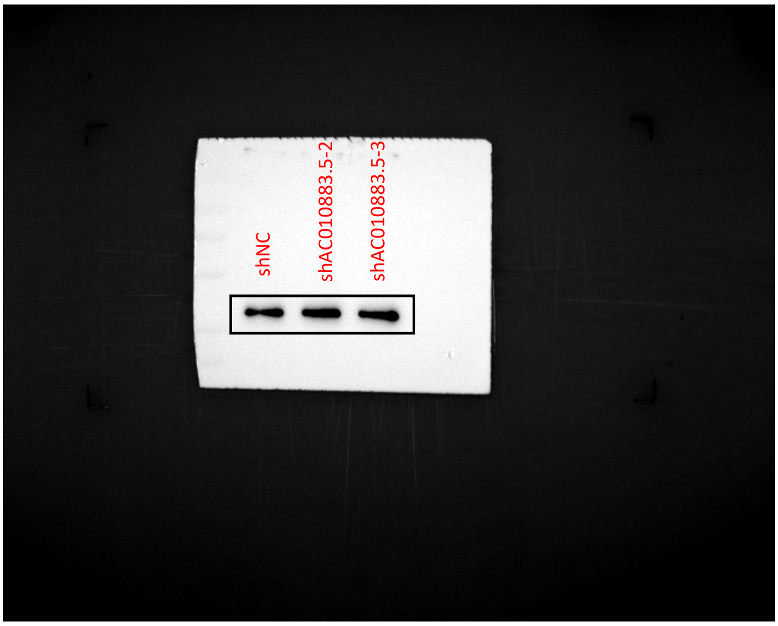
shNC
shAC010883.5-2
shAC010883.5-3

GAPDH

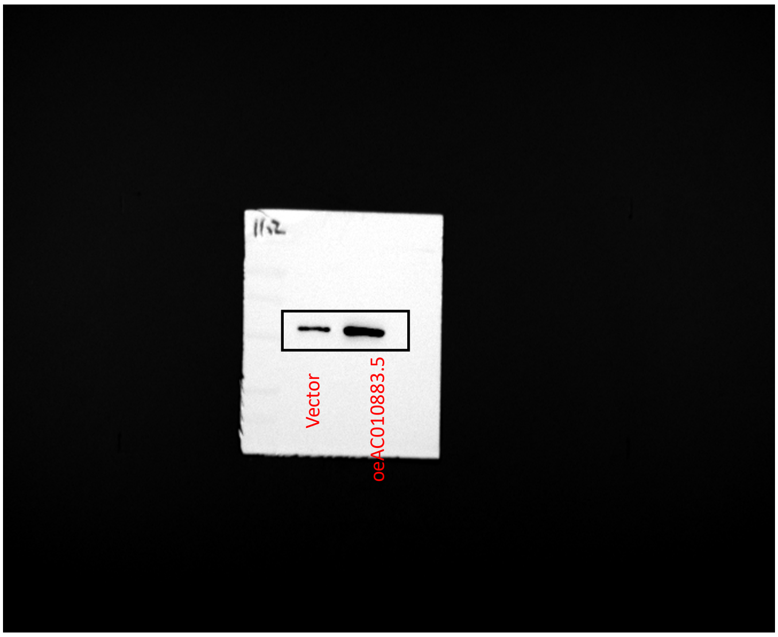

p-MEK1/2

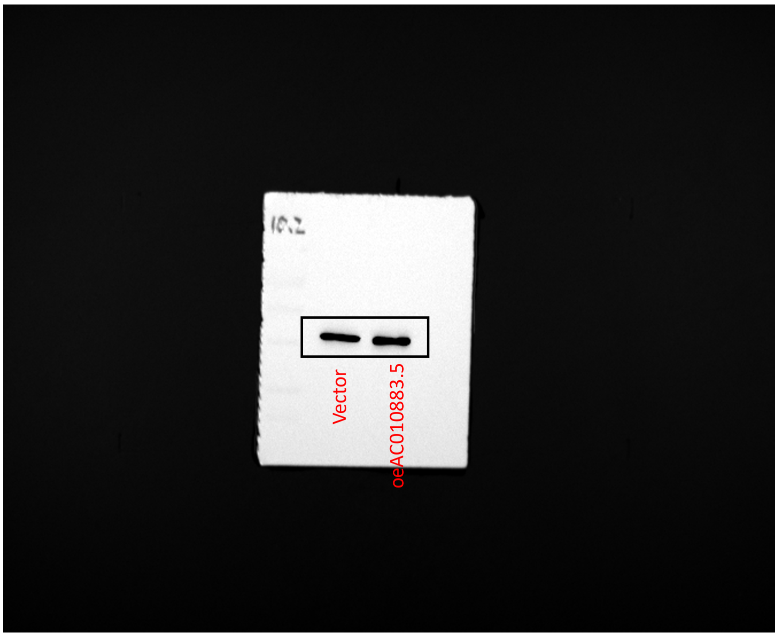

MEK1/2

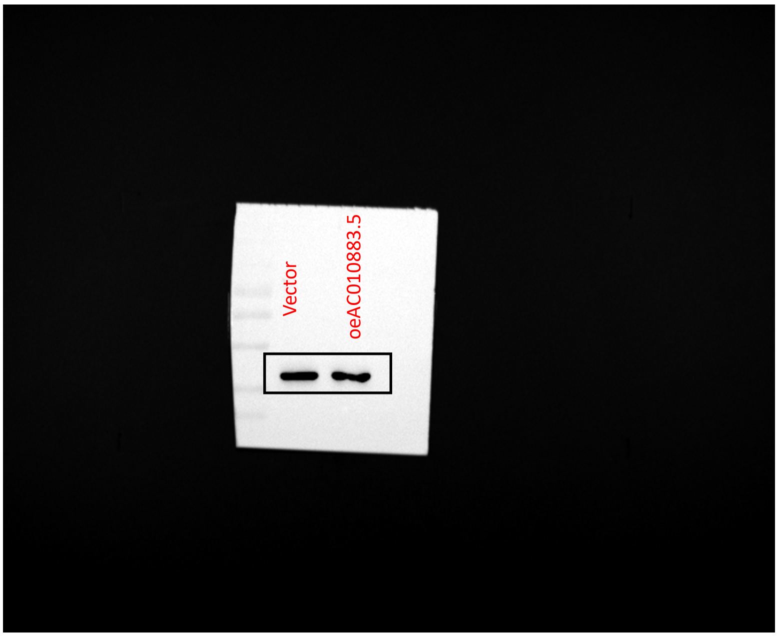

GAPDH

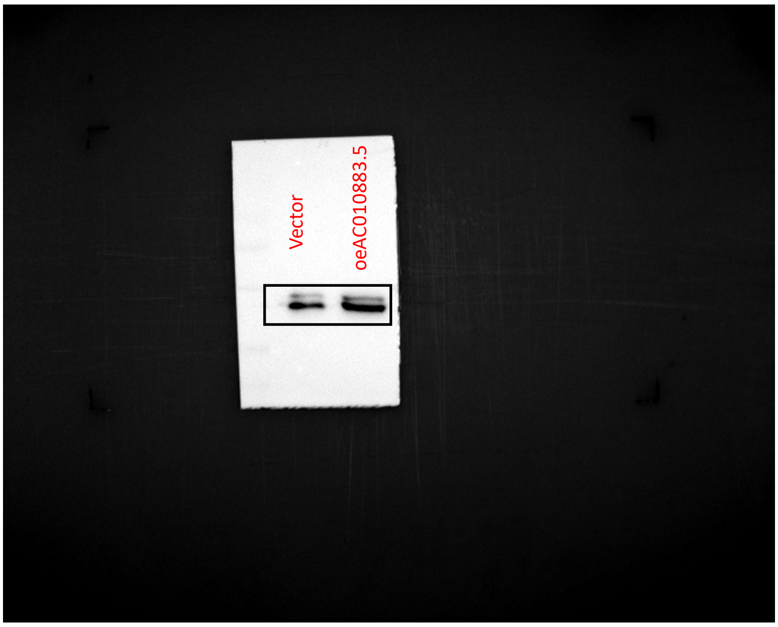

p-ERK1/2

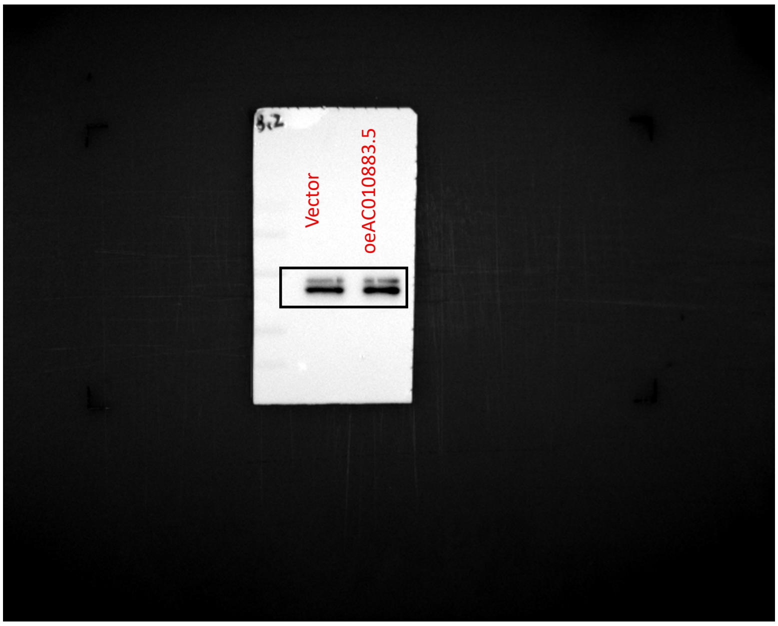

ERK1/2

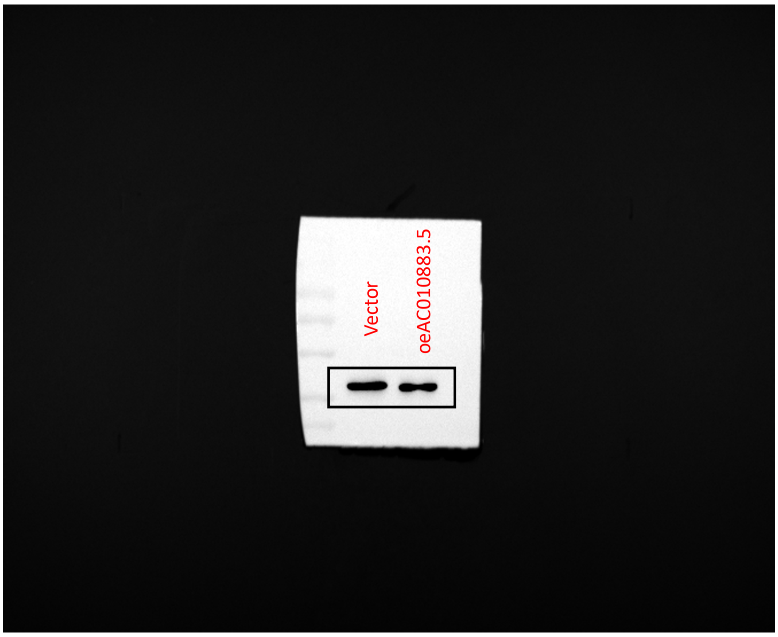

GAPDH

The black boxes show the cropped edge.

The original blots shown in the revised Figure 4I were listed as follow:

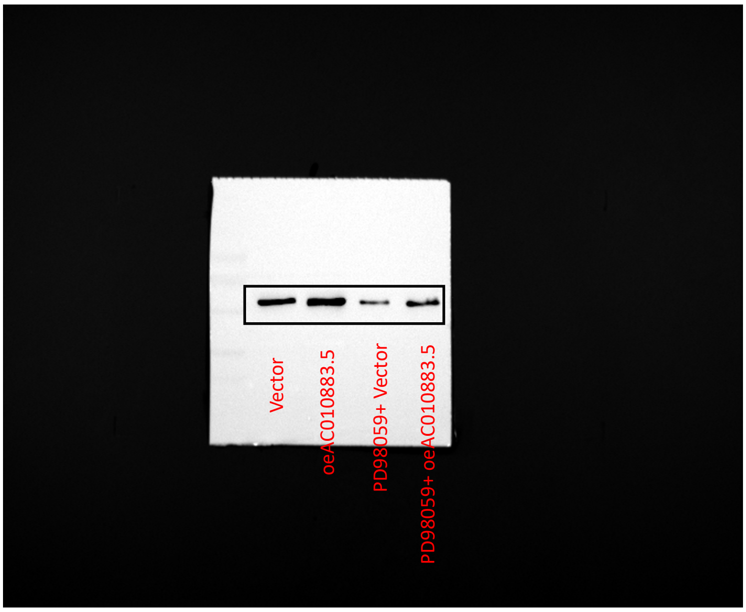

p-MEK1/2

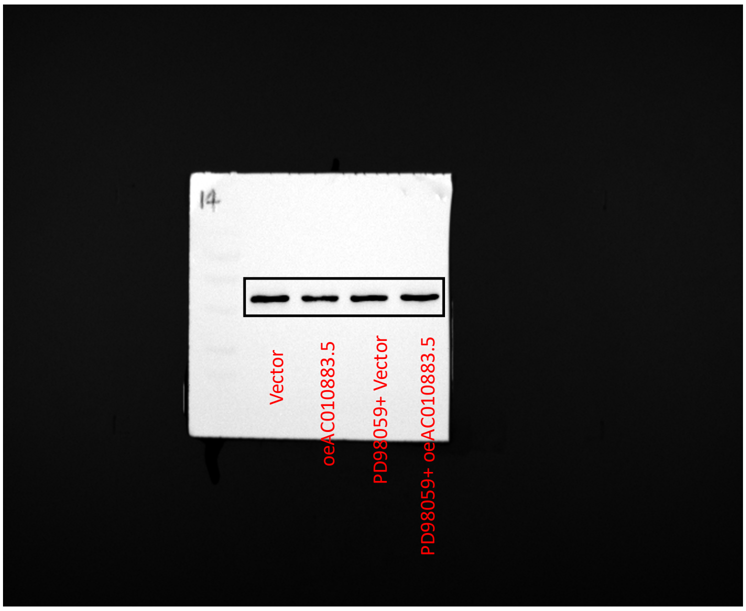

MEK1/2

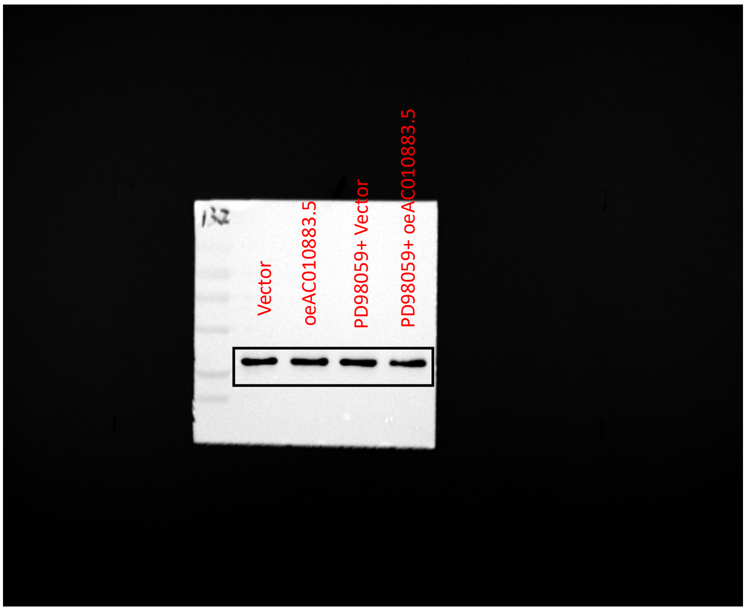

GAPDH

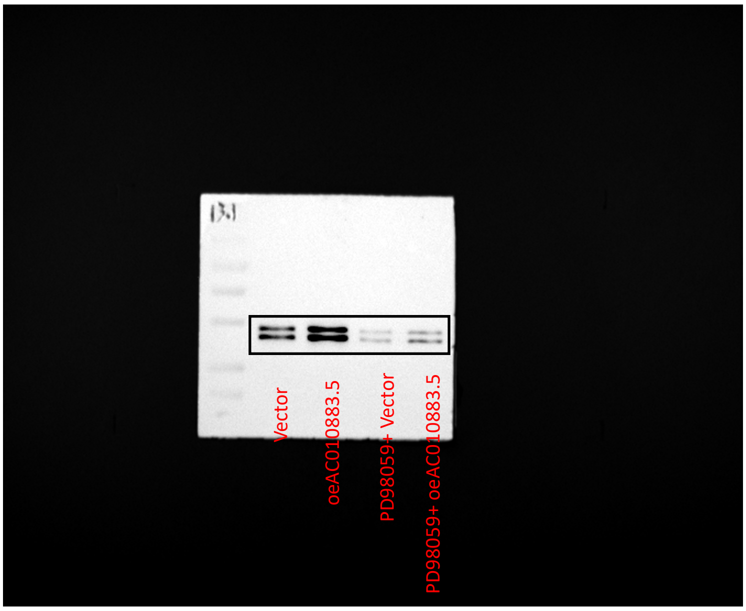

p-ERK1/2

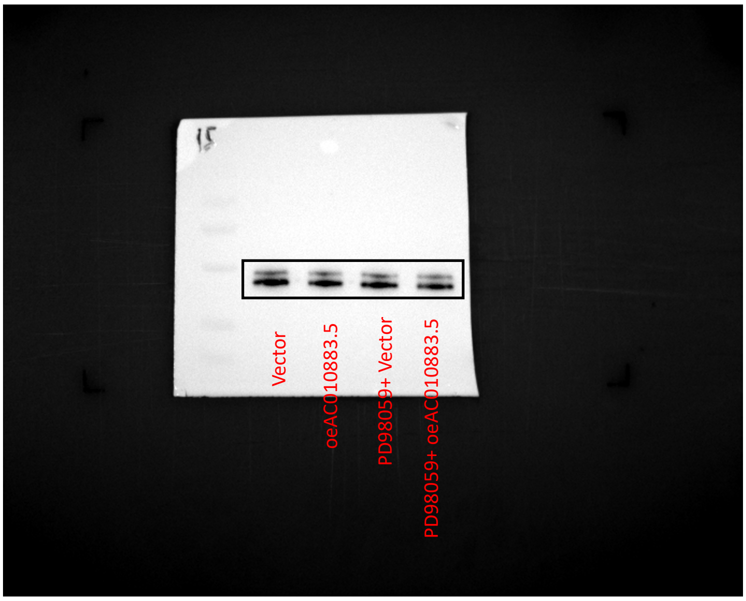

ERK1/2

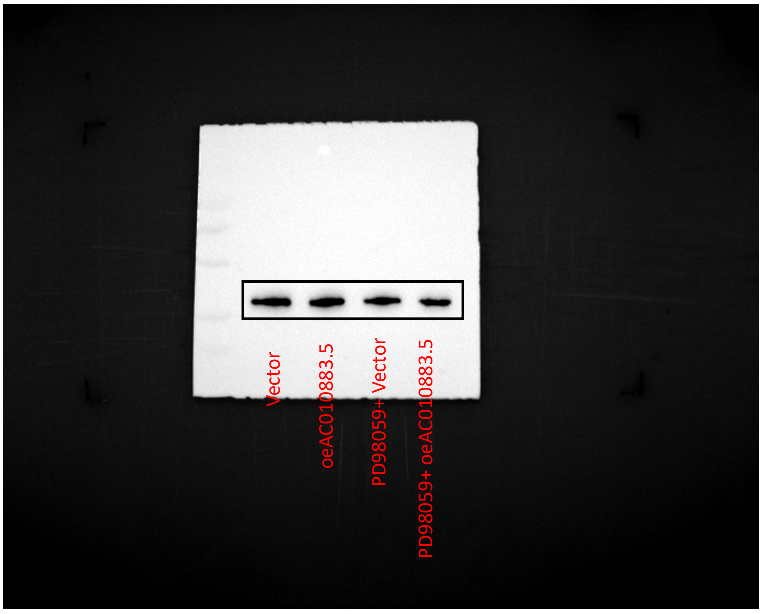

GAPDH

The black boxes show the cropped edge.

The original blots shown in the revised Figure 5H were listed as follow:

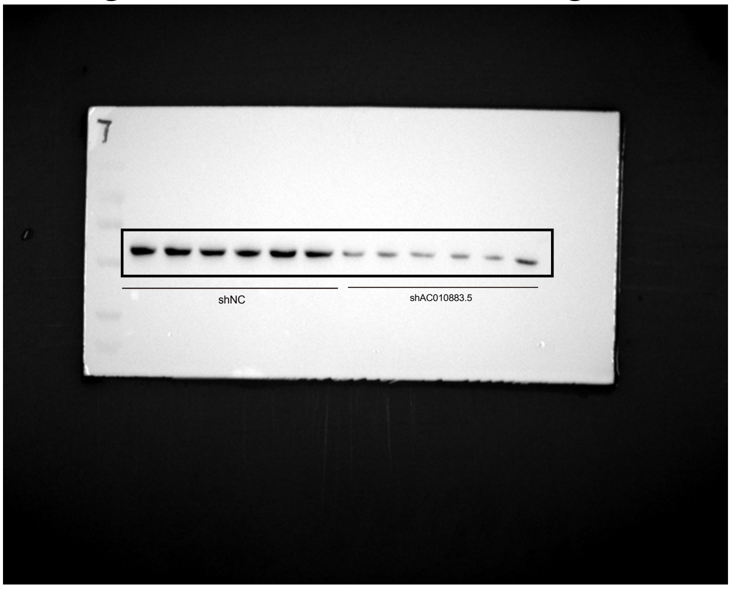

p-MEK1/2

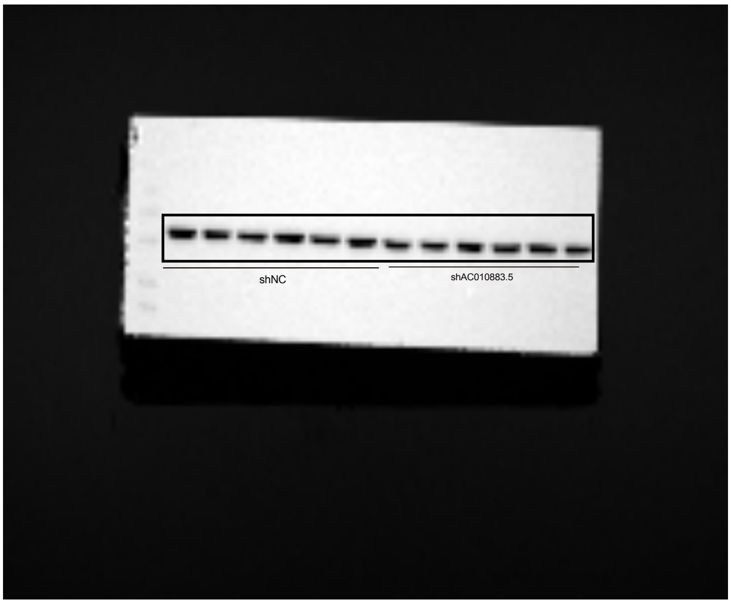

MEK1/2

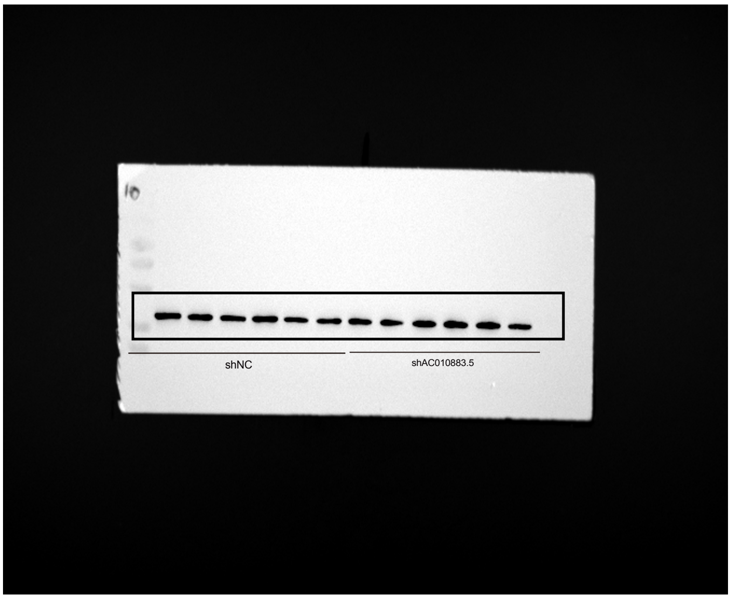

GAPDH

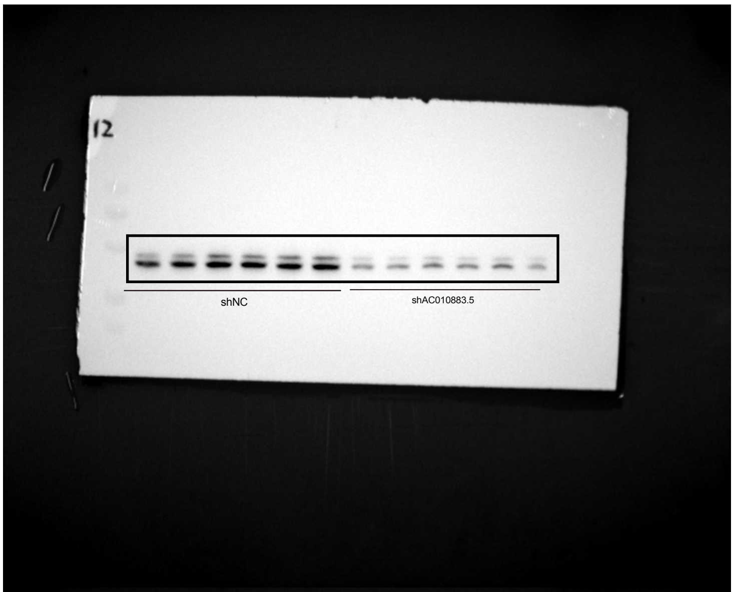

p-ERK1/2

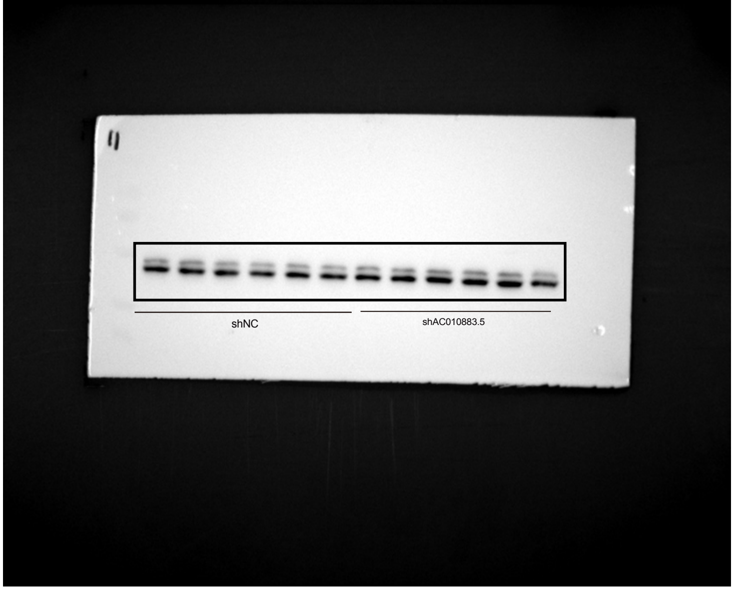

ERK1/2

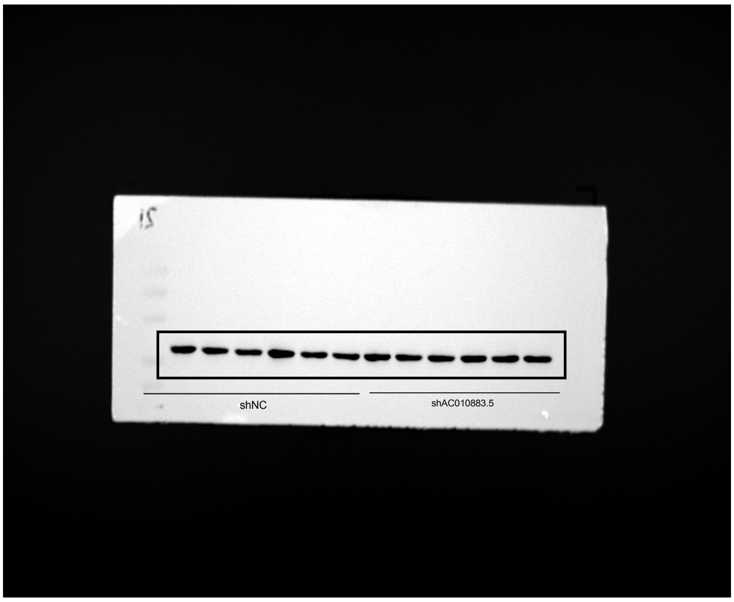

GAPDH

The black boxes show the cropped edge.
